# Photocurable Foam for Three-Dimensional-Printed Porous Structures

**DOI:** 10.1021/acsami.4c10858

**Published:** 2024-08-19

**Authors:** Der-Yun Cheng, Wen-Chien Tai, Ying-Chih Liao

**Affiliations:** Department of Chemical Engineering, National Taiwan University, Taipei 10617, Taiwan

**Keywords:** digital light processing (DLP), foam, porous structures, 3D printing, UV curing, lightweight structures

## Abstract

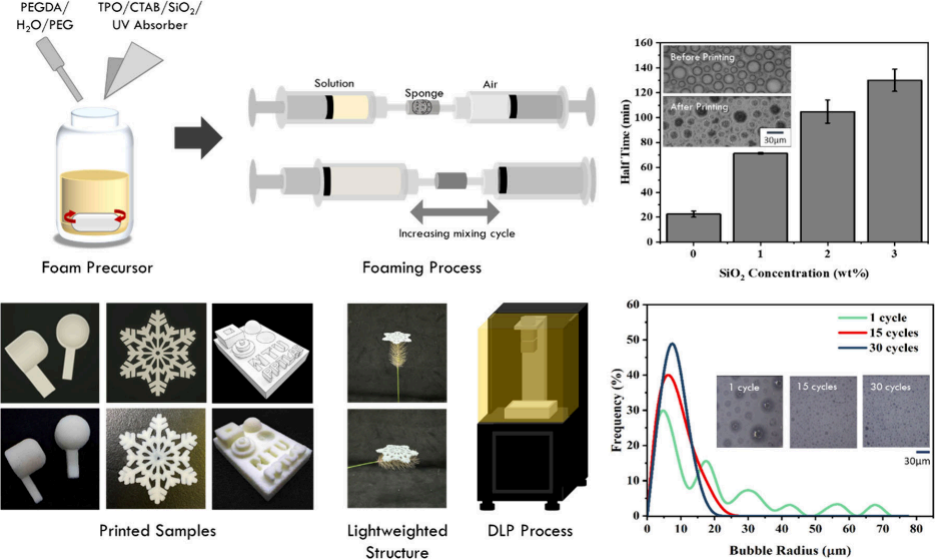

In this research, a foam three-dimensional (3D) printing method using digital light processing (DLP) technology was developed to fabricate 3D-printed porous structures. To address the challenges in preparing DLP precursor foam fluid, we designed a specialized foaming device. This device enables the precursor solution to be blended with air, resulting in a stable foam precursor with an adjustable air/liquid fraction and suitable fluidity, crucially enhancing the gas–liquid contact time for the printing process. By manipulation of fluid flow rates, cycle counts, and gas/liquid ratios, one can easily prepare uniform foams with precise control over the pore size and porosity. To avoid significant volume reduction during ultraviolet (UV) curing, nanoparticle fillers were introduced into the network to prevent collapse of the foam structure. Furthermore, the inclusion of an UV absorber enhanced the quality of the printing process by addressing the limitations associated with particle scattering and reflection. The DLP process can readily fabricate intricate structures, featuring a planar resolution below 30 μm and a printing accuracy of less than 1%. Several examples were also demonstrated to highlight the advantages of this technology and its ability to directly print custom foam structures, thereby saving time and material resources.

## 1 Introduction

Porous structures have been widely used in various industrial applications, such as thermal insulation materials, dust control, noise resistance, and other applications.^1−5^ The most effective method to make porous structures is introducing gas into materials, resulting in low material consumption and lightweight structures. The mechanical properties of these porous structures can also be adjusted by varying the gaseous contents, offering versatility in design to meet specific requirements. Traditional methods for porous or foam structure formation, such as cutting^6^ or injection molding,^7^ often result in significant material waste or prolonged mold fabrication, making them less suitable for customized structures. Instead, three-dimensional (3D) printing provides an efficient solution for constructing these intricate structures with minimal material usage.

To date, many researchers have engaged in 3D printing of porous materials. The most commonly used 3D printing method is the direct ink writing (DIW) technique, which easily constructs 3D structures by extruding viscous inks through a needle and controlling its movements in *x*, *y*, and *z* directions. The shear-thinning properties of inks also help maintain high viscosity during extrusion, preserving structural integrity and allowing for rapid photopolymerization for solidification.^8^ By incorporation of fillers or particles into the inks, a 3D-printed porous structure can be created through post-processing methods, such as chemical treatments (e.g., acid etching^9^) or physical methods (e.g., heating, pressurizing,^10^ or gas injection^11^). Furthermore, in a recent study, Lee et al. innovatively introduce foams into their printing materials by vigorous mixing and produce 3D structures using DIW technology. The printed structures are then solidified through freeze drying and exhibit good mechanical strength.^12^ Rastogi et al. combine foam formation and printing by injecting air and photocurable resin into a nozzle via a Y-shaped tube, extruding foam, partially curing it with ultraviolet (UV) lamps before complete curing upon landing on the substrate.^13^ To further enhance printing resolution, UV curing lithography using a high internal phase emulsion (HIPE) has also been employed. Previous studies have demonstrated successful layer-by-layer UV polymerization of concentrated emulsions.^14,15^ However, due to the high viscosity of HIPE, external forces, such as tilting stages or manual refilling, are needed to ensure uniform coverage of UV-curable material for printing adjacent layers. While these methods show success in creating foams for 3D-printed structures, an additional post-process is commonly needed. Moreover, the filament printing mechanism limits the printing speed of DIW processes, and the printing precision can only provide sub-millimeter resolution due to the nozzle size constraint. Therefore, it remains challenging for one to produce foam structures more rapidly with a fine resolution. Digital light processing (DLP) also offers faster printing speeds and accuracy than DIW with reduced shear force on ink, minimizing foam deformation.^16^ When polyethylene glycol diacrylate (PEGDA) hydrogel is printed with DLP, 3D porous structures can be produced by freeze drying.^17^ Moreover, a polymer with inherent porosity^18^ can also be printed to provide lightweight structures.

To address the challenges in printing and post-processing, the DLP technique offers a viable alternative. Although DLP offers a multitude of advantages, its applicability to porous or foam structures is constrained by the operational characteristics of the DLP machines. There are two main challenges for DLP printing porous structures, namely, light scattering effects and reflowability. Foam precursor solutions are rarely employed in the DLP process because DLP typically requires a transparent polymeric liquid for the photo-curing process, while foam is a blend of air and liquid with low light transmission. The uneven or insufficient UV light exposure of the foam solutions in the printing process can result in bad printing precision and compromises the quality of interlayer bonding. Besides, to enable layer-by-layer printing in DLP machines, the photocurable inks must possess low viscosity, preferably below 5 Pa s, to ensure an expedited and smooth ink reflow process beneath the printing platform. However, to effectively achieve foam stability, foams are designed to possess inherent thin-film structures with low fluidity. Therefore, it is technically difficult to prepare low-viscosity foam solutions with high stability that can resist constant deformation stresses within a DLP machine. These challenges collectively act as substantial hurdles to printing intricate 3D porous structures by directly using foam solutions in DLP 3D printing machines.

In this study, we develop an effective foam generation method for stable and printable foams to enable accurate 3D printing of a porous structure using the DLP process. With a proper surfactant formulation,^19^ a basic foam solution is first produced with a controllable air fraction by vigorous air/liquid mixing. In addition to foam size reduction using higher shear rates,^20,21^ a uniform foam distribution in foam materials^22−25^ is proposed to reduce disparities in foam sizes for lower viscosity with prolonged foam stability.^26^ Therefore, a cyclic foaming mechanism is designed to make the air–liquid mixing gradually converge toward a minimum foam size for the viscosity requirement for DLP machines. To further enhance the printability, the foam precursor solution is also formulated with nanoparticles and UV-curing agents to enable accurate 3D printing of lightweight foam structures with robust mechanical properties. Several examples will be showcased to illustrate the advantages and capabilities of this technology for direct printing of custom foam structures without the need of cutting, assembly, or post-processing steps.

## 2 Experimental Section

### 2.1 Materials

Poly(ethylene glycol) diacrylate [PEGDA, molecular weight (MW) of 700] and hexadecyl trimethylammonium bromide (CTAB, >98%) were purchased from Alfa Aesar, Ward Hill, MA, U.S.A. Fumed silica (SiO_2_) particle powders were purchased from Degussa, Evonik Industries, Piscataway NJ, U.S.A. Diphenyl(2,4,6-trimethylbenzoyl)phosphine (TPO) was purchased from Double Bond Chemical, Taiwan. Eversorb BL3 was purchased from Everlight Chemical, Taiwan. Polyethylene glycol (PEG) with an average MW of 200 was purchased from Sigma-Aldrich, St. Louis, MO, U.S.A. All chemicals were used without further purification.

### 2.2 Preparation of an UV-Curable Foam Precursor

**Figure 1:**
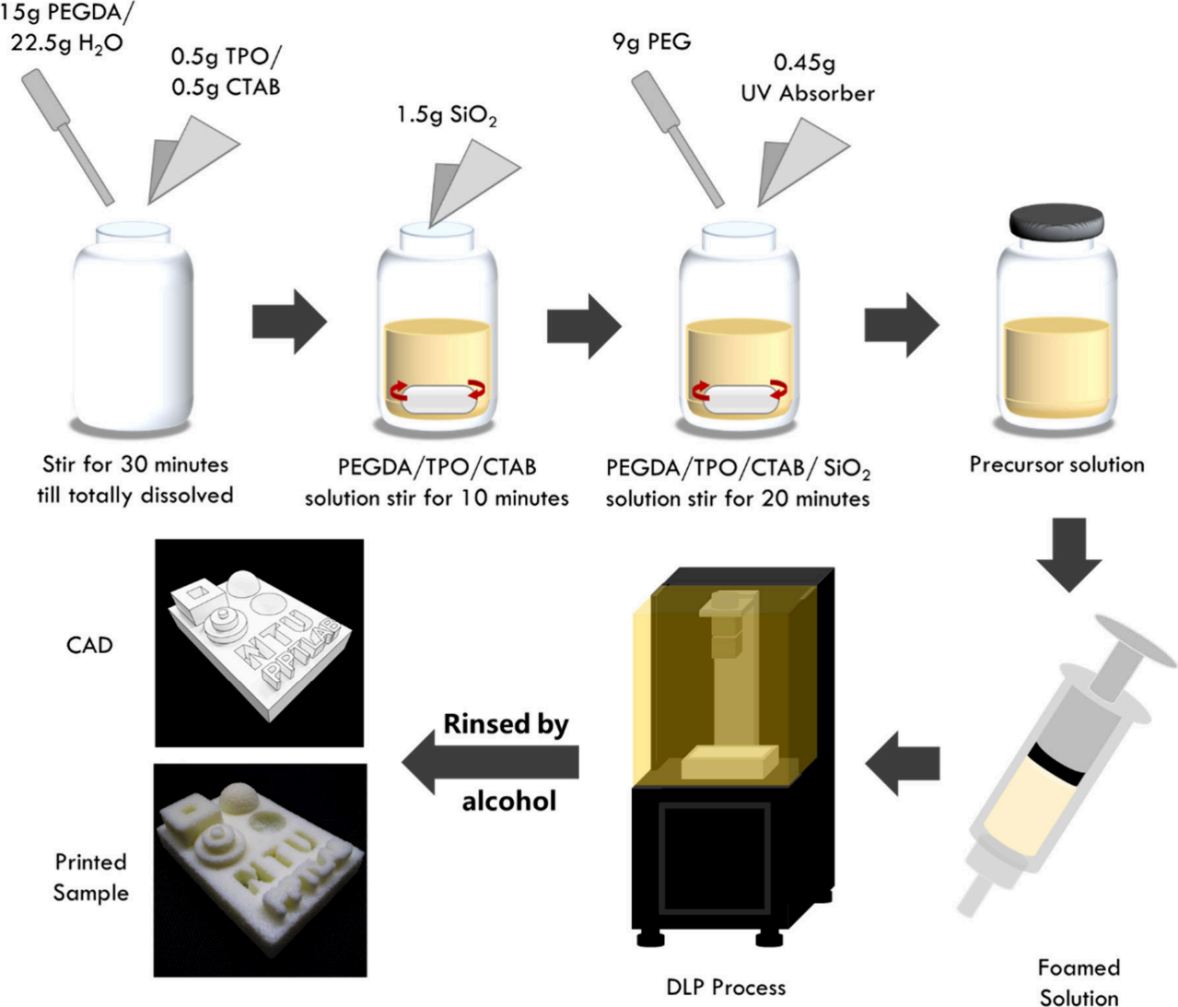
Illustration of the ink preparation and printing process.

The procedure is illustrated in Figure 1. First, 15 g of PEGDA was mixed with 0.5 g of photoinitiator, TPO, and stirred by a magnetic stirrer until the powder was totally dissolved. Then, 22.5 g of deionized (DI) water were added to the solution. Subsequently, 0.5 g of CTAB and 1.5 g of fumed silica were added and stirred for 20 min at 150 rpm. Afterward, 9 g of PEG and 0.45 g of UV light absorber (BL3) were added and mixed for 10 min until the powder dispersed uniformly. All of the above experimental steps were operated under room temperature and ambient pressure.

### 2.3 Foaming Apparatus Design

**Figure 2:**
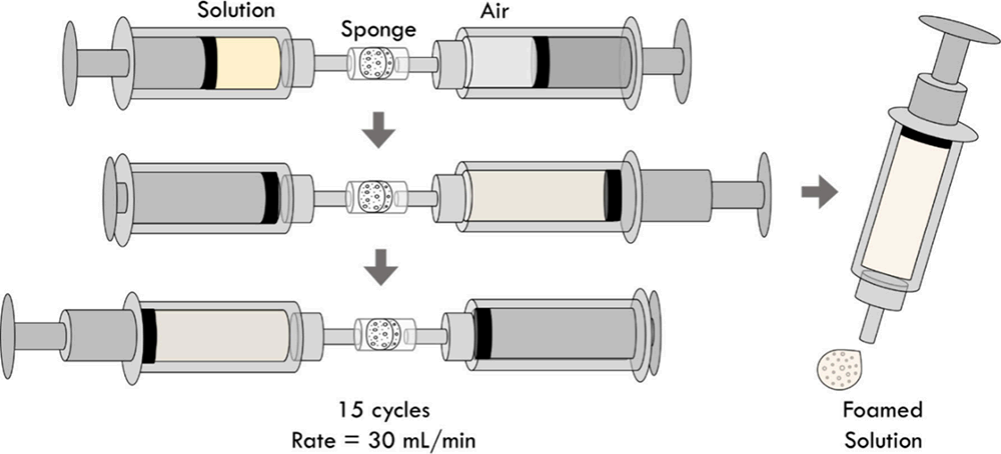
Schematic diagram of the cyclic foaming device.

The foam generation setup is composed of two syringes of the same size (5 cm^3^). Air was drawn into syringe A in predetermined quantities, while syringe B was used to extract ink samples with a precise amount. The two syringes were connected using a Luer lock adapter, creating a sealed space where a sponge with specific pore sizes (100 and 300 μm) was placed. Foam generation was achieved using a syringe pump (NE-400, New Era Pump Systems, East Farmingdale, NY, U.S.A.), operating at different speeds (5, 10, 15, 20, 25, and 30 mL/min) to introduce air into the solution. This process was repeated multiple times to ensure the thorough mixing of air and solution. The visual representation of this setup can be found in Figure 2.

### 2.4 Characterization

The foams were observed under an optical microscope, and the foam size was determined using a commercial software, ImageJ (National Institutes of Health, Bethesda, MD, U.S.A.). The rheological properties of the foams, including viscosity, storage and loss moduli, yield stress, and thixotropy, were measured by a rheometer (Discovery HR-2, TA Instruments, New Castle, DE, U.S.A.), which was equipped with a cone–plate with a diameter of 40 mm and a cone angle of 3.5949°. The viscosities of samples were measured at shear rates ranging from 0.01 to 350 s^–1^. The modulus was measured between angular frequencies of 0.06 and 60 rad/s. All measurements were performed at 25 °C. Scanning electron microscopy (SEM, JEM-2100F, JEOL, Japan) was used to analyze the size and shape of prepared samples. Surface tension of the liquids was measured using an in-house goniometer.

## 3 Results and Discussion

### 3.1 Design of the Foaming Device

**Figure 3:**
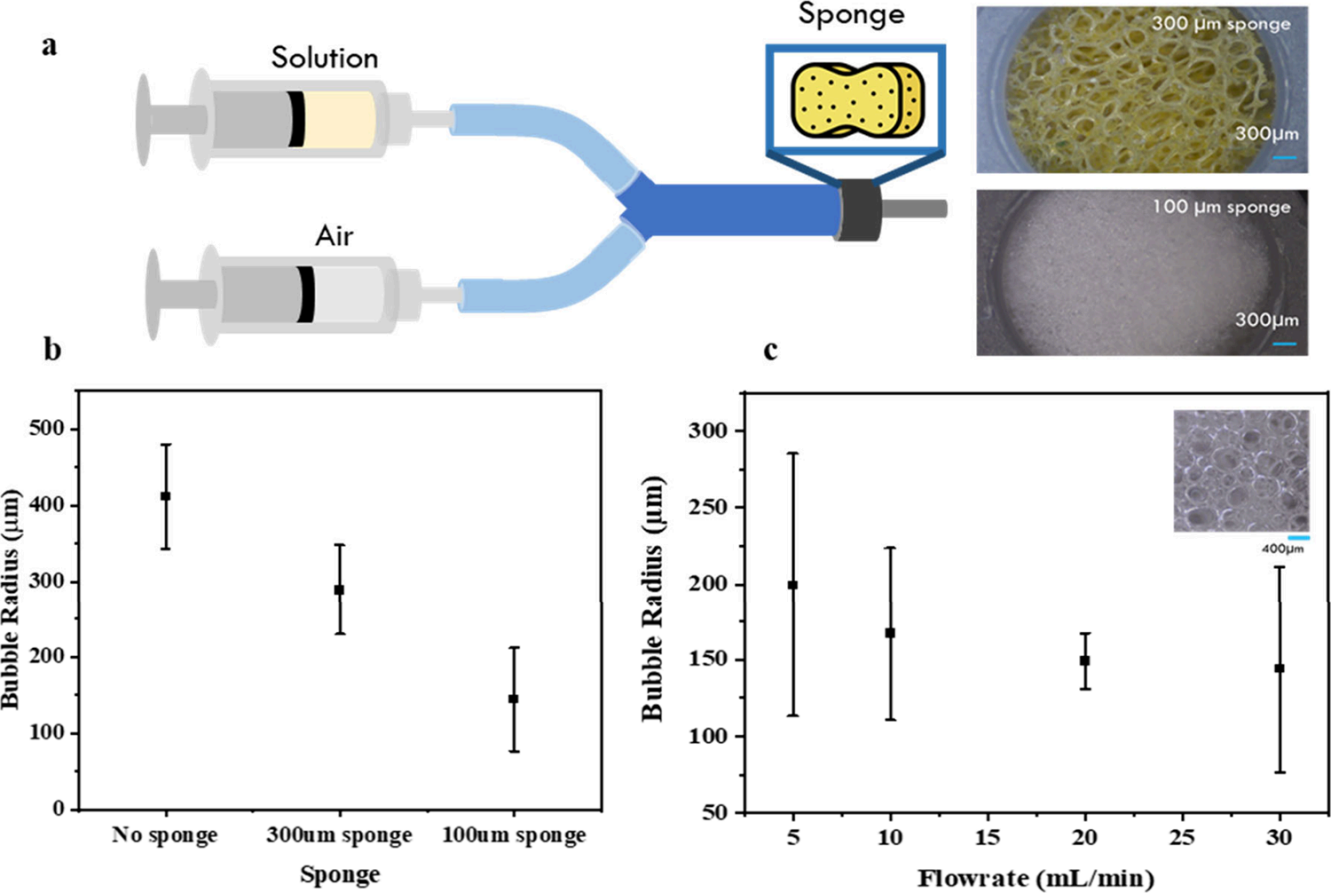
(a) Schematic diagram of a Y-shaped tube device for foam generation. (b) Variation of the average foam radius with sponge pore sizes. (c) Average foam radius as a function of air flow rates.

The foam formation process is first studied to create stable and uniform foams by mixing air and liquid solutions. A two-syringe setup, one containing air and the other containing liquid solution, is initially employed (Figure 3a). A foam liquid solution is first prepared by mixing 30 wt % PEGDA, 1 wt % TPO, and 1 wt % CTAB. As shown in Figure S1 of the Supporting Information, at a low CTAB concentration, the surface tension of the mixture is around 66 mN/m and decreases to 46 mN/m when the addition of CTAB reaches 1 wt %. With the large surface tension differences between the surfactant additions, foams can be generated easily by hand shaking the liquid mixture. To produce small and uniformly sized foam, a high shear rate is needed when the air is introduced into liquids.^27^

The air/liquid blending for foam generation is facilitated by connecting the two syringes with a Y-shaped tube with controllable volumetric flow rates to precisely control the air/liquid ratios. To provide necessary shear force for foam formation and to regulate foam size, a porous sponge is placed at the tip of the Y-shaped tube (Figure 3a). As the air and liquid solution travel through the sponge, the air/liquid mixing occurs to generate foams. As indicated in the literature, the foam size can be reduced with a higher shear rate^20^ with a smaller sponge pore size.^27^ Therefore, when a smaller sponge pore size is used, a finer foam is obtained with a smaller average foam radius (Figure 3b). Notably, despite the size reduction, the foam exhibits a significant size variation. Thus, as one increases the flow rate for air/liquid mixing, the average size remains nearly the same (Figure 3c). The foam sizes are significantly large, exceeding the required printing precision of 25 μm. Consequently, a more effective foaming device for better foam size reduction and uniformity is needed.

**Figure 4:**
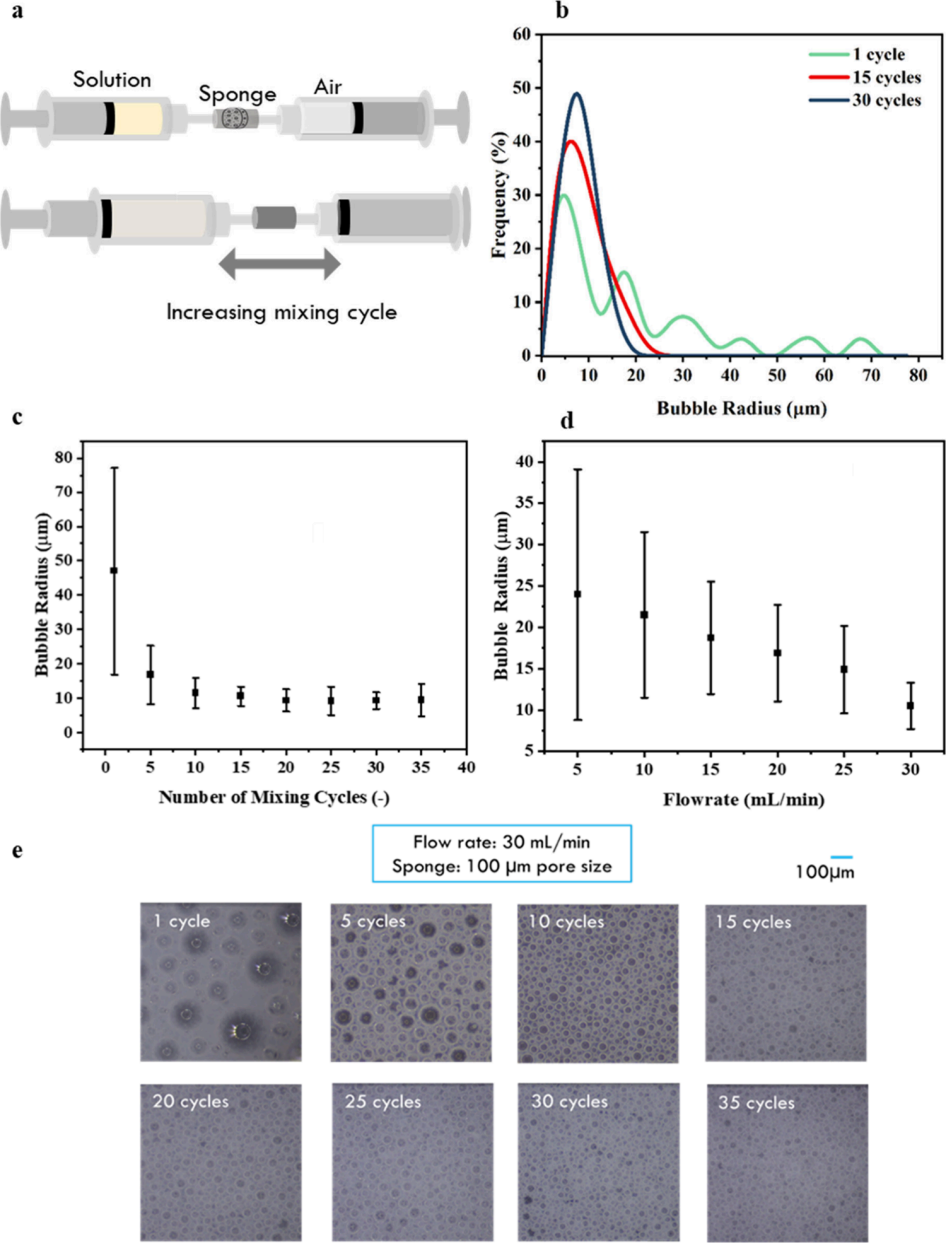
(a) Schematic diagram of the cyclic foam generating device. (b) Foam size distributions after different foaming cycles. Variation of foam size with the (c) number of foaming cycles and (d) flow rates. The error bars show the standard deviation in the foam size distribution. (e) Optical images of foams after different foaming cycles.

To facilitate the foam size reduction in the foaming process, a cyclic foaming approach is utilized to prolong the mixing duration (Figure 4a). The two syringes, one filled with air and another filled with liquid, are connected directly with a sponge in between. When the mixture is pushed back and forth, subsequent mixing can be achieved with multiple cycles. As shown in Figure 4b, in the first cycle, the generated foam has multiple peaks and a wide variation in size. After more circulation cycles in the foaming apparatus, a significant reduction in the foam size can be observed. Finally, after 15 cycles, the foam shows only one peak with a normal distribution. Moreover, as shown in Figure 4c, the foam size distribution remains nearly unchanged after 15 cycles, indicating convergence toward a minimum foam diameter. Different from the previous case in Figure 3, with 15 foaming cycles, the foam size becomes smaller and more uniform as the pumping flow rate increases (Figure 4d) due to the higher shear forces generated in the foam formation process. The average foam size converges after 15 cycles in this device (Figure 4e) with a uniform, fine foam radius of approximately 10 μm. From these findings, a flow flow rate of 30 mL/min with 15 cycles is employed in the subsequent sections to efficiently generate foams while minimizing foam size.

### 3.2 Formulation Adjustments for Printability

**Figure 5:**
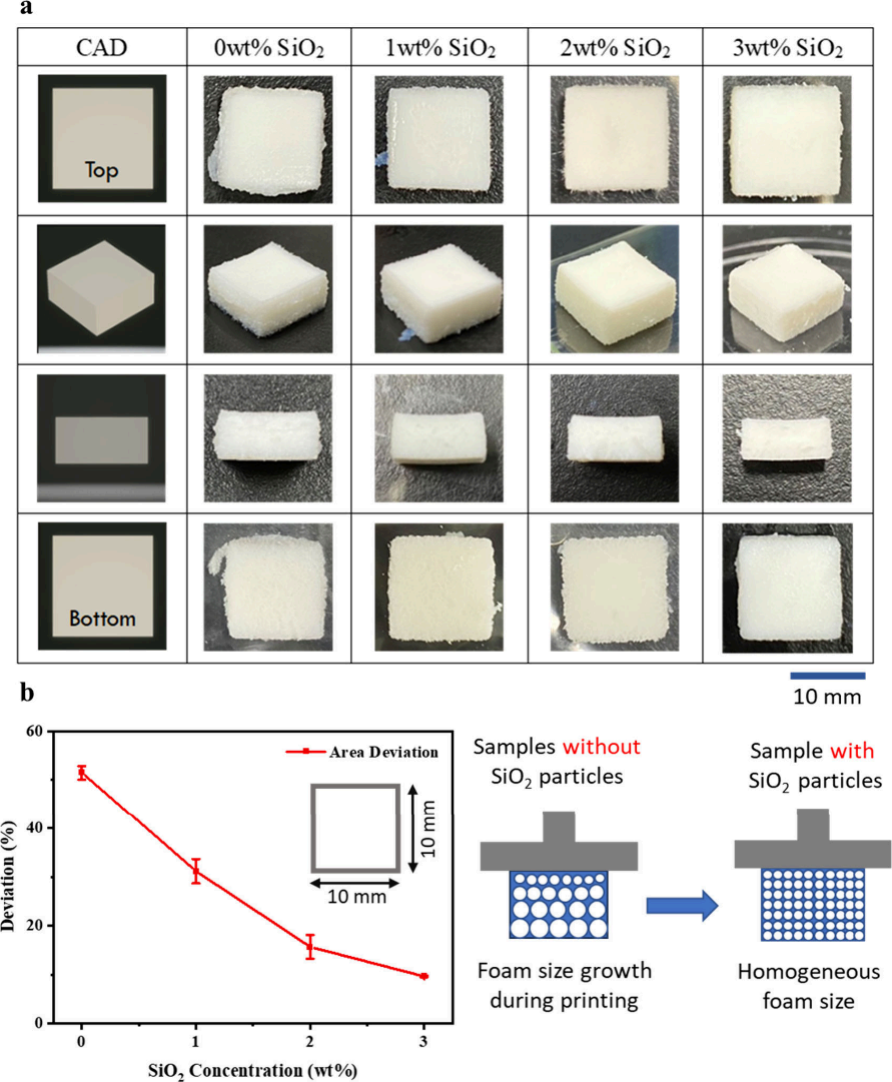
(a) Images of samples from different view angles with varying SiO_2_ concentrations. (b) Influence of SiO_2_ nanoparticle addition on the print quality and foam stability when printing samples with 60% air content.

**Figure 6:**
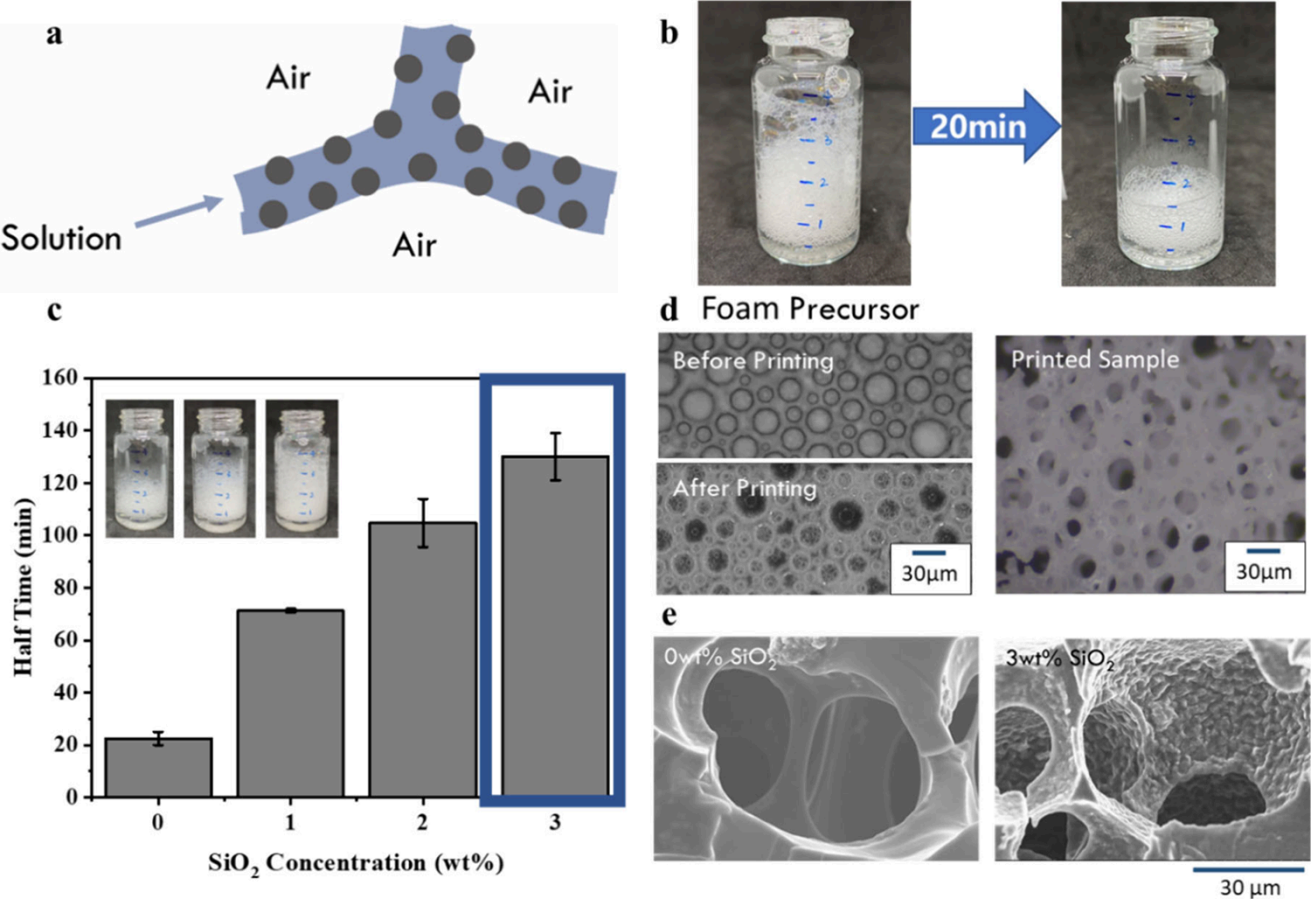
(a) Illustration of particle adsorption at the air–solution interface. (b) Fast reduction of an unstable foam. (c) Stability (half time) of foams at various SiO_2_ concentrations. (d) Pictures of the foam precursor before and after printing. (e) SEM images of printed foam structures without and with SiO_2_ (3 wt %).

Besides dimensional stability for structural size control, the foam precursor also needs strong foam stability to prevent warpage during the printing process. As observed in Figure 5a, the precision of the printed cubes improves with the addition of 3 wt % SiO_2_ compared to those without SiO_2_ particles. In the left panel of Figure 5b, the area variation is minimized with the addition of 3 wt % SiO_2_. To enhance the foam stability, a thickening agent, SiO_2_ nanoparticles, is added in the precursor fluid. As shown in the schematic diagram (the right panel in Figure 5b), the addition of SiO_2_ ensured that a consistent foam size is maintained throughout the DLP printing process.^21^ As shown in Figure 6b, the height of the pristine foam stored in a bottle reduced to half after about 20 min. This half time^28^ can be largely increased 120 min after the incorporation of 1 wt % CTAB and 3 wt % SiO_2_ nanoparticles (Figure 6c), indicating the enhanced foam stability and film strength. The enhanced foam stability helps to keep the foam size consistent throughout the printing process. Therefore, the printed porous structures show little warpage and low variation in foam size after wall structure reinforcement with SiO_2_ addition. As shown in Figure 6d, the foam diameter prior to printing ranged from 15 to 50 μm. After being printed, the remaining foam precursor exhibits nearly the same size. Moreover, the pore sizes in the printed structure also show nearly the same sizes. This suggests that the pore size and distribution of the printed items can be well-controlled in the foam generation process with the addition of SiO_2_ nanoparticles. To further study the microstructure of the printed foams, the printed items are freeze-dried and examined with SEM. As shown in Figure 6e, foams without SiO_2_ nanoparticles display a smooth inner surface, while those with 3 wt % SiO_2_ show a wrinkled texture, attributed to the adhesion of particles within the interfoam films. However, when more than 3 wt % SiO_2_ is added, the viscosity of fluid increases to a level unsuitable for DLP printing. Remarkably, the mechanical properties of the printed materials remain unaffected by additional SiO_2_.

**Figure 7:**
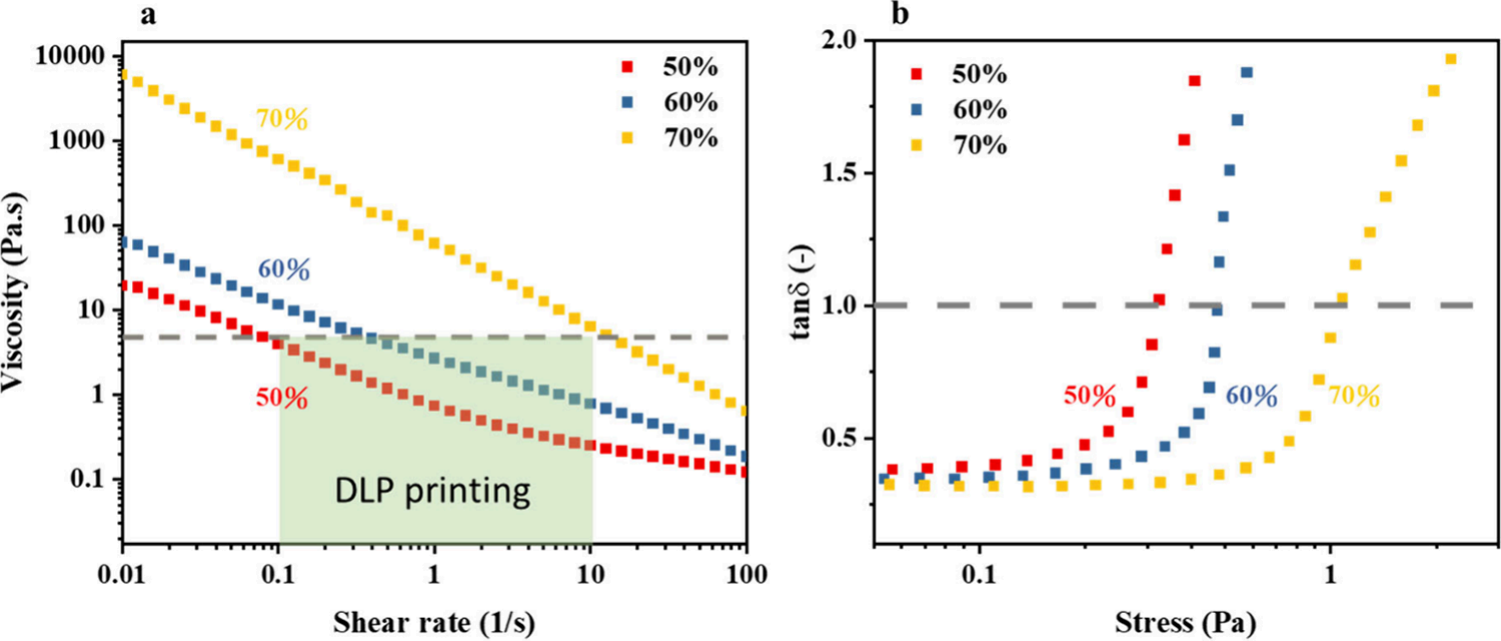
(a) Variation of viscosity with the shear rate for foam samples of different air contents. (b) Variation of tan δ at different air contents (50, 60, and 70%) with shear stress.

The effect of the air fraction in the foam is first investigated to evaluate the printability of the foams in the DLP 3D printing process. In this cyclic foaming mechanism, the air content in the foam can be regulated by changing the gas and liquid contents in the two syringes. As the air content increases, the walls between foam become thinner and, thus, result in larger fluidic resistance in the foam motion. Therefore, as shown in Figure 7a, the viscosity generally increases with the air content and exhibits a shear-thinning behavior. Furthermore, the foam with 70% air content exhibits slow movement, resembling a gel-like nature when the bottle is flipped, highlighting the excessively high viscosity caused by the confined foam.^29^ From examination of the viscoelastic properties (Figure 7b), the foam exhibits nearly solid-like behavior at rest, and thus, the tangent of the phase angle (tan δ) or the ratio between storage and loss moduli is much lower than unity. As the applied stress increases, the friction between foam is lower due to the lubrication of liquid motion, leading to a higher tan δ value. Then, the foam transitions to liquid-like behavior (tan δ > 1) after a critical stress and shows excellent fluidity. For this particular DLP machine, the manual suggests that the viscosity of the printed material needs to be less than 5 Pa s at a shear rate of 10 s^–1^ to avoid printing defects in the DLP reflow process. Therefore, considering the required ink properties for DLP systems, the air content is set at 60 vol % in the following sections.

**Figure 8:**
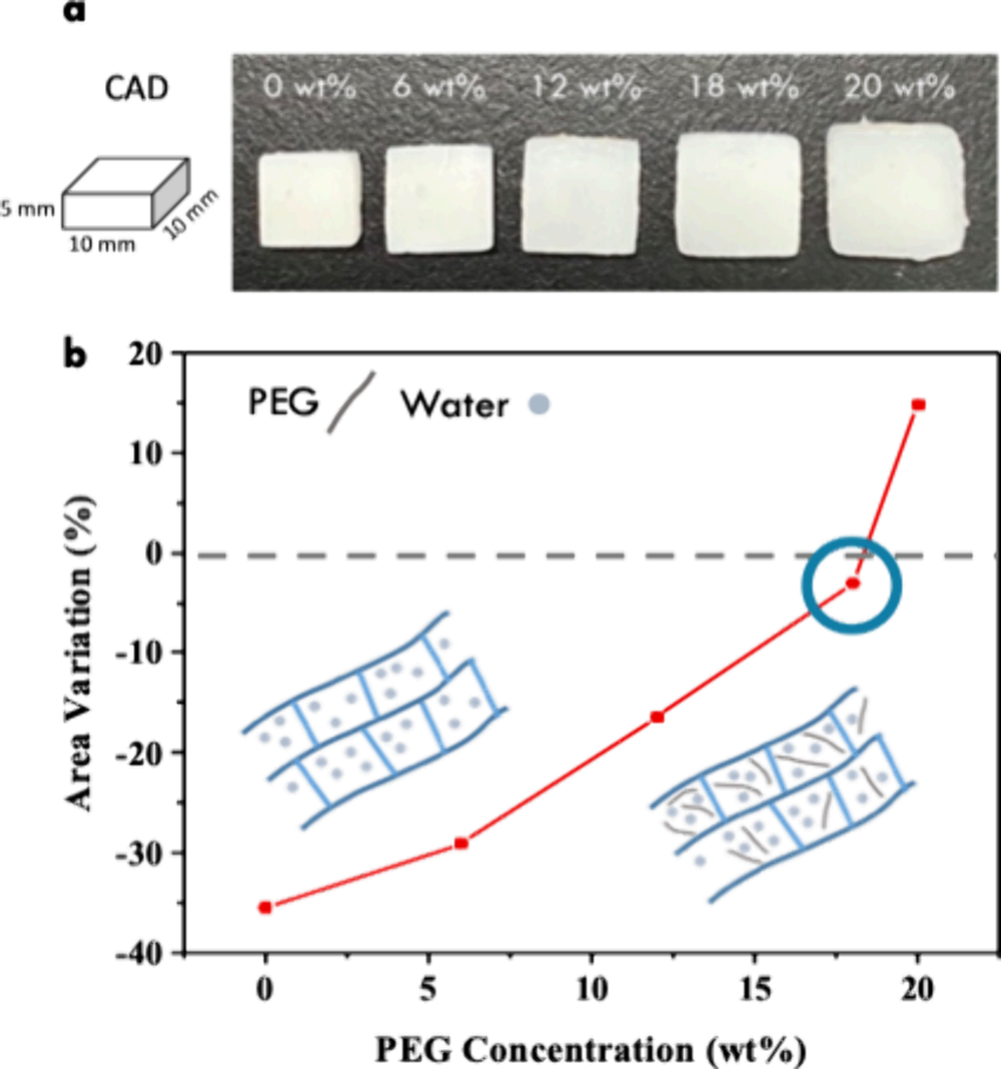
(a) Dimensional control after the addition of PEG. The images show the top view of the printed samples, and the pristine sample is noticeably smaller than those with PEG addition. (b) Area variation of printed samples with the PEG concentration.

Besides the viscosity requirements, the dimensional stability is also of critical importance for 3D printing quality. The relevant printing parameters are shown in Figure S2 of the Supporting Information. In the printing process, the cross-linking precursor usually leads to higher density or volume shrinkage after photopolymerization. This is especially problematic in 3D-printed foams, where excessive shrinkage can distort and weaken porous structures. As shown in Figure 8a, not only are significant changes in sizes observed (Table S1 of the Supporting Information), but defects and cracks are also observed in the printed samples. These defects are attributed to potent internal forces driving contraction in the polymer chains. To address this issue, PEG is introduced into the precursor solution as a supportive element.^30^ The incorporation of PEG in a homogeneous mixture with water remarkably enhanced resistance to shrinkage during photocuring, significantly bolstering the structural integrity.

**Figure 9:**
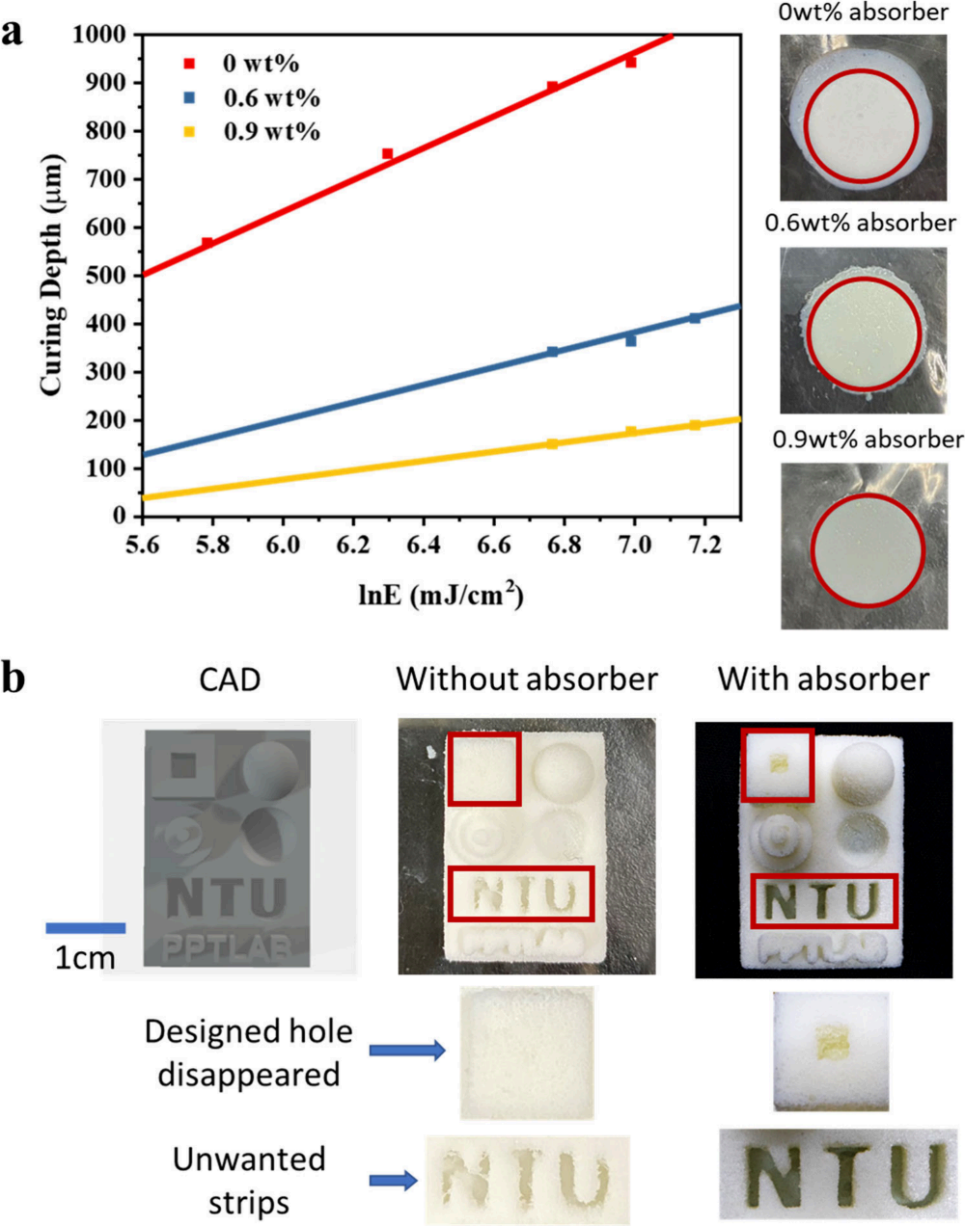
(a) Variation of the curing depth at different light absorber contents (0, 0.6, and 0.9 wt %) with natural log energy of the light sensor. (b) Comparison of 60% foam samples with complex designs before and after the addition of an absorber.

To further acquire the best printing accuracy, an UV absorbing agent is added to control light scattering issues in the DLP printing process. The existence of nanoparticles and foam scatters UV light during the printing process, leading to uncontrolled geometry that is cured by the scattered light irradiation. To tackle this issue, an UV light absorber (BL3 from Eversorb) is introduced to absorb UV light, mitigating refraction and scattering problems to improve the printability.^31^ As shown in Figure 9a, the addition of the absorber decreases the curing depth but largely improves the printing accuracy. With a absorber concentration of 0.9 wt %, the dimensional accuracy can be controlled well within 1% in the *x*–*y* plane, enabling the printing of intricate structures with great accuracy. The addition of an UV absorber also helps the prinnting accuracy in the *z* direction as well. As shown in Figure 9b, deep trenches with a high aspect ratio (depth/gap) can be well-presented in the printed strcutures. These evidence show the possibility of applying the foam precursor for complicated 3D foam structures with good accuracy.

### 3.3 3D-Printed Foam Structures

**Figure 10:**
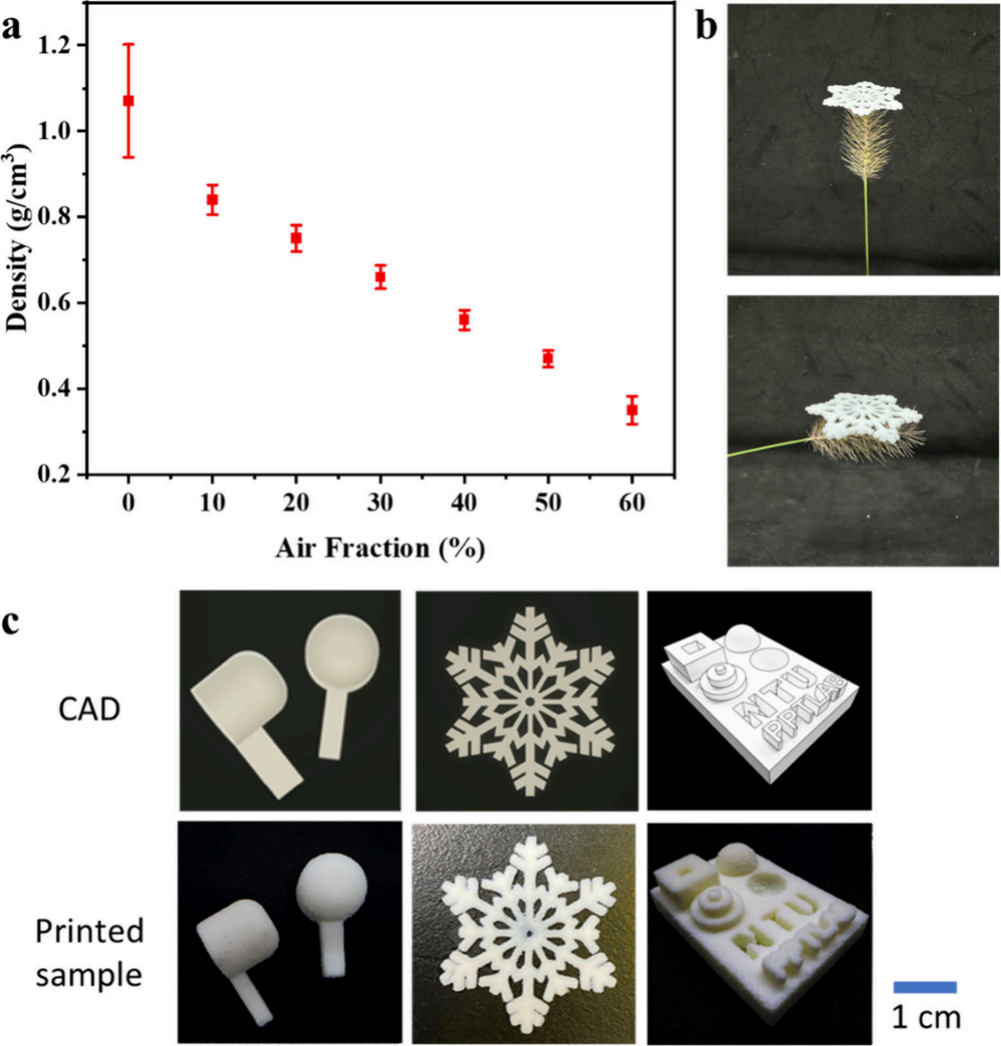
(a) Density of foam samples at different air contents. (b) Placement of a printed snowflake-patterned foam structure on foxtail grass. (c) Comparison between various 3D-printed products and their CADs. The samples in the presented images are made of inks with 60% air content.

With this foam ink for the DLP 3D printing process, one can quickly create various intricate 3D porous structures. As shown in Figure 10c, headphone plugs, snowflakes, and many other 3D geometries can be accurately printed with ease and show good accordance with the original computer-aided designs (CADs), demonstrating the versatility of this 3D foam printing process. This printing method also provides remarkably linear density control (Figure 10a). By adjusting the air contents in the foaming device, one can easily change the porosity and achieve a maximum 60% weight reduction compared to solid objects. With the weight reduction, this foam printing method can therefore provide an effective tool for lightweight designs. As shown in Figure 10b, when a printed snowflake pattern is placed on top of the foxtail grass, the grass supports the printed snowflake pattern effortlessly without deformation, demonstrating the remarkably low weight nature of the printed structure. With the great printing accuracy and porosity control, this 3D foam printing method offers a promising manufacturing tool to generate functional porous structures with a convenience for various applications.

After successful 3D printing, efforts are also made to explore its potential applications in daily life, with a particular focus on its mechanical properties. Compression strength tests were performed on cubes measuring 10 × 10 × 5 mm. The initial foam precursor exhibited a compression strength of approximately 1.8 MPa after curing, indicating satisfactory support strength when compared to other hydrogels.^9,32−34^ As shown in Figure S3 of the Supporting Information, an increase in the air content led to a decrease in both compression strength and Young’s modulus, while the compressive strain at fracture increased. By adjustment of the air content, the mechanical properties of foam structures can be tailored to meet specific requirements, enabling versatile design and customization.

## Conclusion

In this study, we present a novel method to directly 3D print foam structures using DLP printing technology. To address challenges in preparing DLP precursor foam fluid, a specialized foaming device is designed to achieve precise control over the pore size and porosity, resulting in uniformly sized foam with a radius of 10 μm. Nanoparticle fillers and an UV absorber are also used to mitigate volume reduction during UV curing and to enhance the resolution of the printing process. The demonstrated capabilities of the DLP process in fabricating intricate structures with a planar resolution below 30 μm and a layer thickness precision below 100 μm showcase its versatility and precision. The porosity of the printed foam structures can be predetermined by adjusting the foam formulation and can reach an apparent density of less than 0.35 g/cm^3^. Several examples are also showcased to demonstrate the direct fabrication of lightweight porous structures without the need for post-processing steps. The cyclic foam-producing device can be further extended to other precursor systems to directly print 3D foam structures. In summary, this research presents a significant advancement in the realm of 3D printing and provides a versatile approach to creating customized foam-based porous structures for various applications.

## Supporting Information Available

Details of experimental procedures, variation of the surface tension at different CTAB concentrations, effects of varied PEG concentrations on printed solutions with 60% air contents, and variation in the area with different surface power densities (PDF)
