# Supplementary material for: Photocurable Foam for Three-Dimensional-Printed Porous Structures
Source: ACS Appl Mater Interfaces. 2024 Aug 19;16(34):45589–97. doi: 10.1021/acsami.4c10858 (PMC11367572; doi:10.1021/acsami.4c10858)
Supplement: Supplementary file 1 — am4c10858_si_001.pdf [file am4c10858_si_001.pdf]

Supporting Information:

Photocurable Foam for 3D-Printed Porous Structures

*Der-Yun Cheng[1], Wen-Chien Tai[1], and Ying-Chih Liao[1]**

[1]Department of Chemical Engineering, National Taiwan University, No. 1, Sec. 4, Roosevelt Rd., Taipei 10617, Taiwan

E-mail: liaoy@ntu.edu.tw

Contents:

1. Variation of surface tension at different CTAB concentrations.
2. Effects of varied PEG concentrations on printed solutions with 60% air contents.
3. Variation in area variation with different surface power density.
4. Mechanical properties of printed samples with different air fraction.

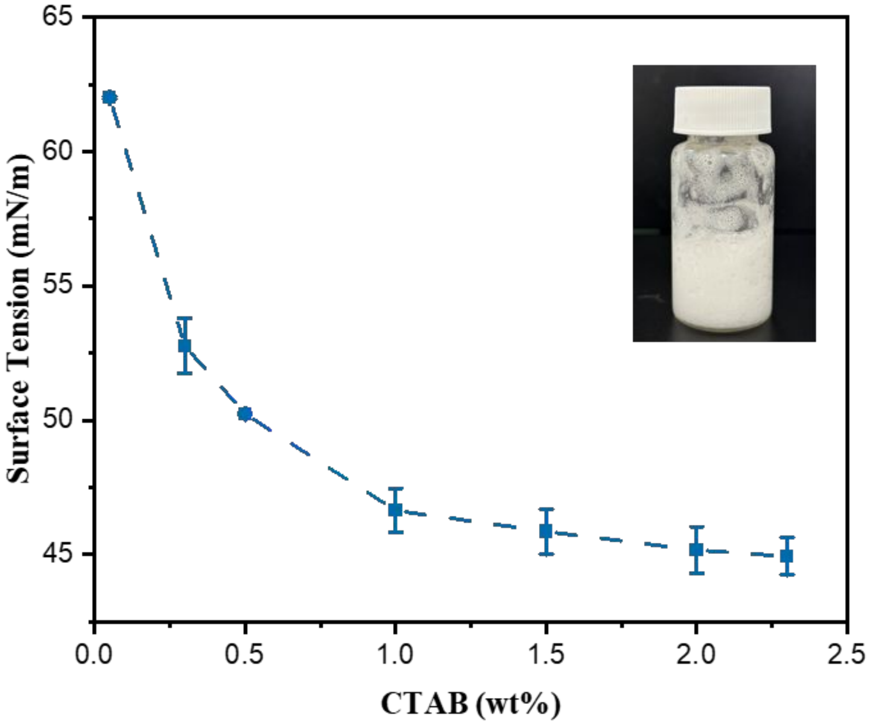

Figure S1. Variation of surface tension at different CTAB concentrations.

Table S1. Effects of varied PEG concentrations on printed solutions with 60% air contents.

PEG conc. (wt%)	0	6	12	18	20
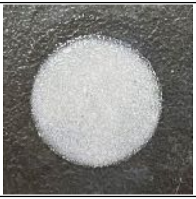	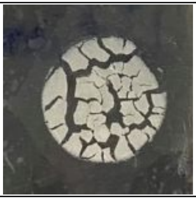	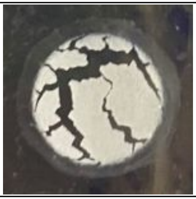	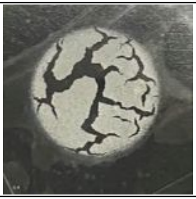	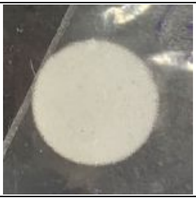	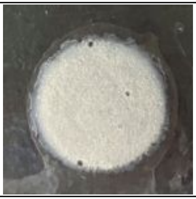
Volume shrinkage (%)	13.74	9.18	7.14	4.65	3.72
Area deviation (%)	-35.50	-29.06	-16.46	-2.97	14.85

Due to the sample shrinkage after photocuring, the effect of PEG concentrations (0 wt%, 6 wt%, 12 wt%, 18 wt%, and 20 wt%) was measured. First, the solution was drawn into a 1 mL syringe, and the mass was measured to determine the density before curing. The uncured samples were then printed into a 10 mm diameter circle. Their weight and volume were subsequently measured. Since the cured samples were not perfectly disk due to shrinkage, the volume was measured using the displacement method, and the density after curing was obtained. The shrinkage rate for the different samples was calculated using an equation based on the density before and after curing.

$$V_s(\%) = \frac{(1/\rho_m)-(1/\rho_p)}{1/\rho_m} \times 100 \qquad (1)$$

where V_s is the volume shrinkage rate, ρ_m is the density before curing, and ρ_p is the density after curing.

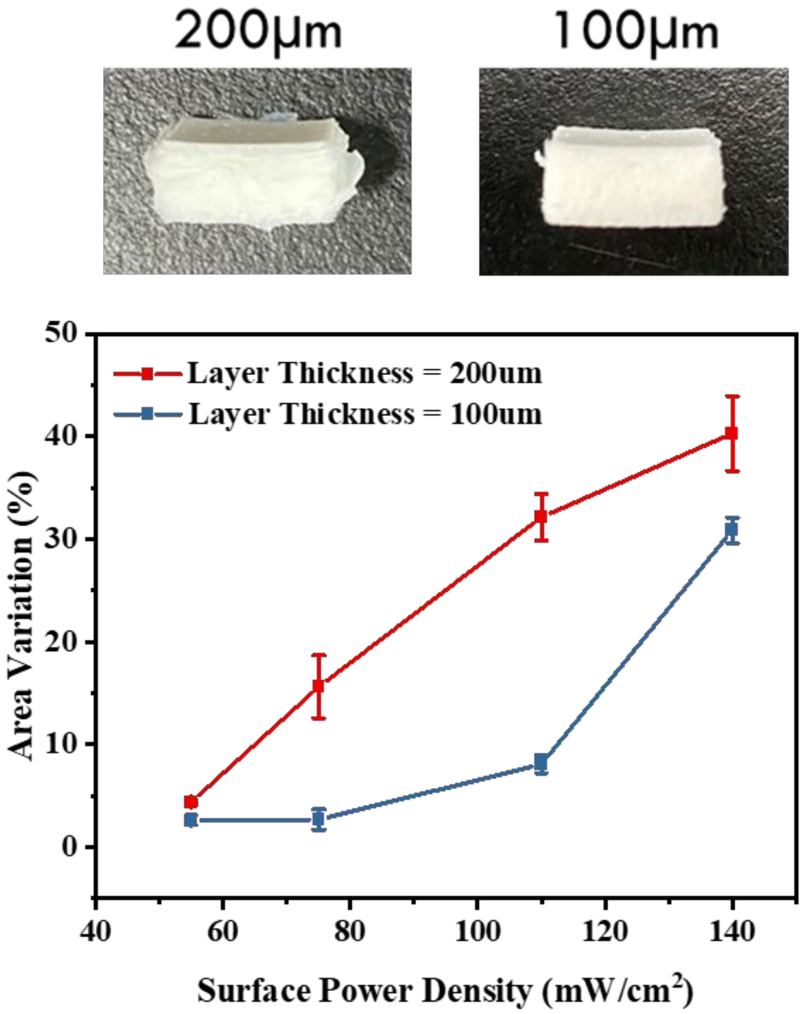

Figure S2. Variation in area variation with different surface power density.

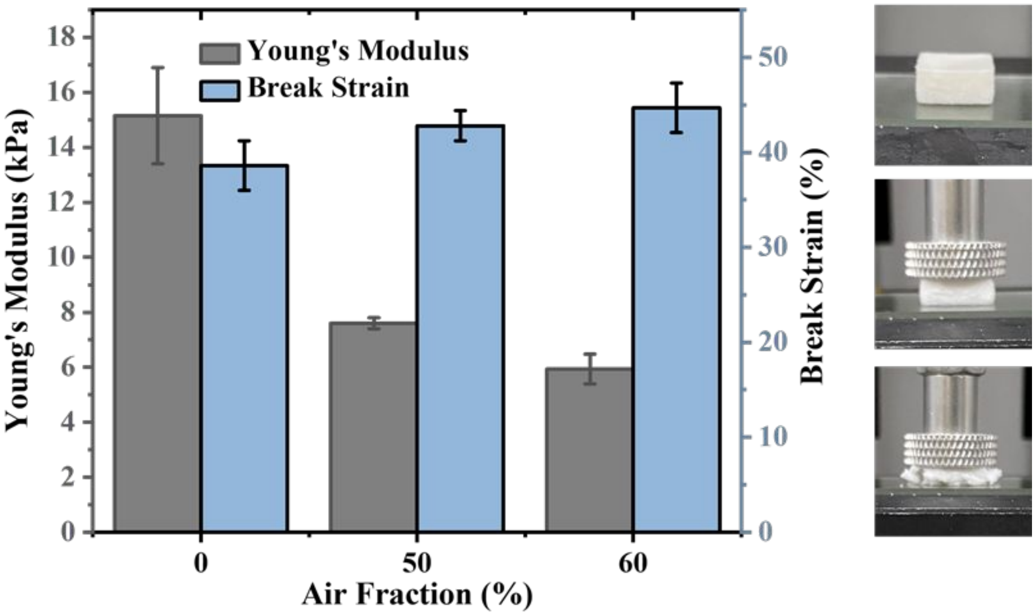

Figure S3. Mechanical properties of printed samples with different air fraction.
